# Effect of a lifestyle intervention during chemotherapy for breast cancer on quality of life

**DOI:** 10.1093/jncics/pkaf125

**Published:** 2025-12-26

**Authors:** Leah S Puklin, Fang-Yong Li, Leah M Ferrucci, Brenda Cartmel, Maura Harrigan, Courtney McGowan, Michelle Zupa, Jennifer A Ligibel, Tara Sanft, Melinda L Irwin

**Affiliations:** Yale School of Public Health, New Haven, CT, United States; Yale School of Public Health, New Haven, CT, United States; Yale School of Public Health, New Haven, CT, United States; Yale Cancer Center, New Haven, CT, United States; Yale School of Public Health, New Haven, CT, United States; Yale Cancer Center, New Haven, CT, United States; Yale School of Public Health, New Haven, CT, United States; Yale School of Public Health, New Haven, CT, United States; Yale School of Public Health, New Haven, CT, United States; Dana-Farber Cancer Institute, Boston, MA, United States; Yale Cancer Center, New Haven, CT, United States; Yale University School of Medicine, New Haven, CT, United States; Yale School of Public Health, New Haven, CT, United States; Yale Cancer Center, New Haven, CT, United States

## Abstract

**Background:**

Chemotherapy-induced side effects can diminish physical and psychological well-being for women with breast cancer. Although nutrition and exercise improve quality of life (QoL) posttreatment, their ability to attenuate treatment-related declines in QoL during chemotherapy remains underexplored.

**Methods:**

Women diagnosed with stage I-III breast cancer initiating chemotherapy were randomized to a yearlong nutrition and exercise intervention (I; *n* = 87) or usual care (UC; *n* = 86). Patient-Reported Outcomes Measurement Information System (PROMIS)-29, PROMIS Cognitive Function, and PROMIS Global Health scales were assessed at diagnosis (baseline), postchemotherapy (PC), 1-year, and 2-years postrandomization.

**Results:**

Participants (*N* = 173) were on average 52.8 ± 11.1 years of age and 51% had stage I breast cancer. At diagnosis, PROMIS scores were comparable to the general US population, except for heightened anxiety. PROMIS scores worsened from diagnosis to PC for physical function (I = −5.5 (1.0); UC = −5.4 (1.0)), fatigue (I = 5.4 (1.1); UC = 6.2 (1.1)), social roles (I = −5.4 (1.0); UC = −7.1 (1.0)), cognitive function (I = −4.8 (1.0); UC = −4.3 (1.1)), global physical health (I = −10.9 (0.8); UC = −10.1 (0.8)), and global mental health (I = −11.2 (1.1); UC = −9.7 (1.2)), with anxiety improving (I = −5.1 (0.9); UC = −3.7 (0.9)). No between-arm differences were observed. By 1 year, most scores returned to baseline levels and remained stable through 2 years, except anxiety, which remained improved.

**Conclusion:**

Despite improving nutrition and exercise, the intervention did not attenuate declines in QoL compared with UC. This study fills a gap on interventions with nutrition and exercise components during chemotherapy and highlights needing more research to identify those most likely to have benefits in QoL from lifestyle interventions delivered during active treatment.

**Clinical Trial Registration:**

NCT03314688

## Introduction

A breast cancer diagnosis often triggers fear, anxiety, depression, and stress.^1^^,^^2^ Chemotherapy can cause fatigue, cognitive impairment, menopausal symptoms, and sleep disturbance, which independently reduce mental and physical well-being and exacerbate the psychological distress of a cancer diagnosis.^3^^,^^4^

Nutrition and exercise interventions in breast cancer survivors have reduced symptom burden, such as fatigue and anxiety, and improved physical and mental health in the posttreatment setting.^5-8^ To date, most trials conducted during active cancer treatment have focused exclusively on exercise and have found sufficient evidence to support exercise during treatment to preserve physical function and reduce fatigue, anxiety, and depression.^9-13^

Nutrition interventions during active treatment are limited, highlighting uncertainty around the effects of dietary intervention during treatment on treatment-related symptoms and physical and mental well-being.^13-16^ Between 31% and 59% of women with breast cancer report experiencing at least 1 nutrition impact symptom, such as nausea, diarrhea, and loss of appetite, during chemotherapy.^17^^,^^18^ Nutrition interventions that support the management of nutrition impact symptoms are critical as they may help maintain nutritional status, preserve muscle mass and strength, and subsequently improve physical and mental well-being during and after treatment. Thus, more evidence is needed to evaluate nutrition interventions during treatment, alone, or combined with exercise, on quality of life (QoL).

The Lifestyle, Exercise, and Nutrition Early After Diagnosis (LEANer) trial randomized 173 women with breast cancer initiating curative-intent chemotherapy to a yearlong nutrition and exercise intervention or usual care to compare the effect on chemotherapy adherence. This secondary analysis examined the effect of the LEANer intervention versus usual care on longitudinal Patient-Reported Outcomes Measurement Information Systems (PROMIS) QoL measures at baseline (diagnosis), postchemotherapy, and 1- and 2-years postrandomization.

## Methods

### LEANer study and participants

The LEANer study and primary outcome (chemotherapy adherence) have been described previously.^17^^,^^19^ Briefly, 173 women newly diagnosed with breast cancer were randomized to a yearlong comprehensive nutrition and exercise intervention (*n* = 87) versus usual care (UC; *n* = 86). Eligible participants included women diagnosed with anatomic stage I-III breast cancer, receiving chemotherapy, exercising <150 min/wk of moderate- to vigorous-intensity physical activity, eating <7 fruits or vegetables daily, physically able to walk, and able to understand instructions in English. Ineligibility criteria included started second chemotherapy cycle, pregnant or planning to become pregnant, stroke or myocardial infarction in the past year, dementia or major psychiatric disease, or participating in a weight loss program.

### Recruitment

Participants were recruited between February 2018 through July 2021 from the Smilow Cancer Hospital Network at Yale-New Haven Hospital and the Dana-Farber Cancer Institute. Eligibility was initially assessed through electronic health records (EHRs), followed by confirmation of chemotherapy receipt and oncologist approval and consent to contact the patient. Patients were then screened for interest and eligibility by study staff. All participants provided informed consent. The Yale Human Investigation Committee and the Dana-Farber/Harvard Cancer Center Institutional Review Board approved this study (ClinicalTrials.gov: https://clinicaltrials.gov/ct2/show/NCT03314688).

### Intervention

The intervention consisted of 16 thirty-minute counseling sessions delivered by registered dietitians (RDs), who were Certified Specialists in Oncology Nutrition by the Academy of Nutrition and Dietetics, with additional training in exercise science. The sessions were completed in-person, by phone, or by video per participant preference and COVID-19 restrictions. Sessions began before the second chemotherapy cycle and lasted 1 year (4 weekly sessions in the first month, biweekly sessions for months 2 and 3, and monthly sessions thereafter). The intervention content was based on the LEAN study,^20^ which was adapted from the Diabetes Prevention Program,^21^ and grounded in the Social Cognitive Theory,^22^ with modifications to address active cancer treatment. The counseling sessions aimed to improve physical activity and dietary behaviors, with an emphasis on managing chemotherapy symptoms.^17^^,^^19^^,^^23^ The exercise goal was to gradually reach 150 min/wk of moderate-intensity physical activity and twice-weekly strength training, aligned with the Physical Activity Guidelines for Americans^24^ and American Cancer Society and American College of Sports Medicine guidelines for cancer survivors.^25^^,^^26^ The nutrition component promoted improving diet quality through a predominantly plant-based diet based on the Dietary Guidelines for Americans and cancer survivorship recommendations.^25^^,^^27^

### Usual care

Women randomized to UC were referred to the Survivorship Clinic, and if they requested a nutrition consultation, they were referred to the hospital dietitian per standard of care. At the end of the trial, UC participants were given study intervention materials and offered a counseling session with the study RD.

### Randomization and blinding

Participants were randomized after completing baseline questionnaires. Randomization was stratified by human epidermal growth factor receptor 2 (HER2) status (HER2 positive or HER2 negative), hormone receptor status (HR) (HR-positive or HR-negative), and number of planned chemotherapy cycles (4 or >4) with computer‐generated randomization lists for each stratum using the permutation method with variable block sizes. The study biostatistician was blinded to group allocation.

### Measures

Sociodemographic data (age, race and ethnicity, education, marital status, employment, insurance, and menopausal status (no menstrual period ≥12 months)) were collected at baseline via online or paper questionnaires. Clinical data (weight, height, anatomic cancer stage using the American Joint Committee on Cancer 7th/8th edition, chemotherapy type and duration, tumor subtype) were abstracted from EHRs.

Physical activity was assessed using a validated, interviewer‐administered questionnaire capturing type, frequency, and duration of activities over the past 3 months.^28^ Dietary intake over the same period was assessed via a food frequency questionnaire from the Fred Hutchinson Cancer Center’s Nutrition Assessment Shared Resource.^29^^,^^30^ The Nutrient Data System for Research software version (2020), developed by the University of Minnesota, Minneapolis, MN, calculated nutrient intake.^29^^,^^30^ Diet quality was calculated with the Healthy Eating Index‐2015 (HEI‐2015), a positive change reflects improved diet quality (range = 0-100).^31^

The PROMIS measures were collected at baseline, postchemotherapy, and 1- and 2-years postrandomization. We included 6 PROMIS-29 v2.1 domains (Anxiety, Depression, Sleep Disturbance, Social Roles and Activities, Physical Function, and Fatigue),^32^ the PROMIS Cognitive Function Short Form 6a,^33^ and PROMIS Global Health, which yield the PROMIS Global Physical Health and the PROMIS Global Mental Health.^34^ Items were scored on a 5-point Likert scale. Higher scores reflect better physical function, social roles, cognitive function, global mental and global physical health, and worse symptoms (anxiety, depression, fatigue, and sleep disturbance).

All PROMIS scores were standardized to a T-score (mean = 50 and SD = 10 in the US general population from the 2000 General US census) using the online Health Measures Assessment Center Scoring Service.^35^ A 3 T-score (3-unit change) was considered clinically meaningful based on validation in cancer populations.^36^^,^^37^

### Statistical analysis

Baseline characteristics were compared by arm using Student *t*-tests, χ^2^ tests, or Fischer exact tests.

The mean baseline to postchemotherapy, 1-year, and 2-year changes in PROMIS measures were compared by arm using a mixed model repeated measures analysis in an intention to treat (ITT) fashion. Linear contrasts were used to obtain changes in PROMIS measures in each arm and differences by arm, while baseline values of the two arms were constrained to be equal. Least square means and 95% confidence intervals (CIs) were estimated.

Effect modification by baseline PROMIS scores was explored via stratified analyses and tested including arm × time × modifier interactions, with modifiers categorized by ordinal scale or median split. Potential modifiers included age (median split), chemotherapy drug (Adriamycin vs other), chemotherapy (adjuvant vs neoadjuvant), cancer subtype, and disease stage (stage I, stage II, stage III).

We combined arms to examine univariate and multivariable associations between demographic and clinical characteristics and changes in PROMIS outcomes. We only report models for PROMIS outcomes with a clinically meaningful (3-T score) change from baseline to postchemotherapy (anxiety, fatigue, social roles, physical function, cognitive function, physical health and mental health). Multivariable models included select demographic variables and variables significant at *P* < .1 in the univariate models. Last, we reported correlation coefficients between changes in health behaviors and changes in PROMIS measures.

All analyses were conducted using SAS Version 9.4 (SAS, Cary, NC) and tests were two-sided, with a *P* value less than .05 significance level for multivariable models.

## Results

Baseline characteristics were similar between study arms (Table 1). Women were, on average, 53 ± 11 years of age, with a mean body mass index (BMI) of 29.7 ± 6.7 kg/m^2^. Most women were non-Hispanic White (71%) and were 64 ± 33 days from diagnosis. Women were diagnosed primarily with stage I breast cancer (51%), with 42% receiving neoadjuvant chemotherapy.

**Table 1.** Baseline characteristics of women in the LEANer trial by study arm (*N* = 173).

Characteristic	Study arm		***P*** ^c^
	Intervention (*n* = 87)^a^^,^^b^	Usual care (*n* = 86)^a^^,^^b^	
Age	52.3 ± 11.3	53.3 ± 10.9	.57
Postmenopausal	48 (55.2%)	46 (53.5%)	.63
BMI (kg/m^2^)	29.5 ± 7.0	29.8 ± 6.6	.80
Self-report race and ethnicity			
Non-Hispanic White	61 (70.1%)	62 (72.1%)	.46
Non-Hispanic Black	11 (12.6%)	14 (16.3%)	
Hispanic	8 (9.2%)	5 (5.8%)	
Asian or Pacific Islander	2 (2.3%)	4 (4.7%)	
Prefer not to answer	3 (3.5%)	0	
Other^d^	2 (2.3%)	1 (1.2%)	
Education level			
Less than college	29 (33.3%)	36 (41.9%)	.25
College and above	58 (66.7%)	50 (58.1%)	
At enrollment employment status			
Unemployed/retired	23 (26.4%)	30 (34.9%)	.25
Part time (<35 h/wk)	14 (16.1%)	9 (10.5%)	
Full time (≥35 h/wk)	38 (43.7%)	32 (37.2%)	
On leave from job	10 (11.5%)	15 (17.4%)	
Prefer not to answer	2 (2.3%)	0	
Living with somone			
Married or living with someone	66 (75.9%)	61 (70.9%)	.46
Living alone	21 (24.1%)	25 (29.1%)	
Health insurance			
Medicaid	4 (4.6%)	7 (8.1%)	.15
Medicare	9 (10.3%)	10 (11.6%)	
Private	65 (74.7%)	67 (77.9%)	
Other	9 (10.3%)^e^	2 (2.3%)^f^	
Time since diagnosis (d)	60 ± 34	68 ± 32	.08
Randomized before 1st chemotherapy cycle	37 (42.5%)	28 (32.6%)	.18
Randomized before 2nd chemotherapy cycle	87 (100%)	86 (100%)	–
AJCC 8th edition cancer stage at diagnosis^g^			
Stage I	45 (51.7%)	43 (50.0%)	.83
Stage II	31 (35.6%)	34 (39.5%)	
Stage III	11 (12.6%)	9 (10.5%)	
Receptor status			
ER/PR+, HER2−	49 (56.3%)	50 (58.1%)	.95
ER/PR+/−, HER2 +	15 (17.2%)	15 (17.4%)	
Triple negative	23 (26.4%)	21 (24.4%)	
Chemotherapy			
Neoadjuvant	41 (47.1%)	32 (37.2%)	.19
Adjuvant	46 (52.9%)	54 (62.8%)	
Number of chemotherapy cycles			
4 (range of weeks: 8-12)	23 (26.4%)	25 (29.1%)	.70
>4 (range of weeks: 12-24)	64 (73.6%)	61 (70.9%)	
Study site			
Yale	63 (72.4%)	59 (68.5%)	.58
DFCI	24 (27.6%)	27 (31.4%)	
PROMIS-29			
Physical function^h^	49.9 ± 8.6	48.8 ± 8.1	.36
Anxiety^i^	54.8 ± 8.3	54.9 ± 9.1	.96
Depression^i^	47.7 ± 7.2	49.3 ± 8.0	.17
Fatigue^i^	49.5 ± 9.8	50.6 ± 9.4	.43
Sleep disturbance^i^	52.2 ± 8.8	53.6 ± 9.2	.31
Social roles and activities^h^	55.0 ± 8.6	55.1 ± 8.7	.90
PROMIS Cognitive Function			
Cognitive function^h^	51.5 ± 10.0	50.9 ± 10.5	.71
PROMIS Global Health			
Global Physical Health^h^	49.6 ± 7.3	48.7 ± 6.9	.40
Global Mental Health^h^	49.9 ± 8.1	51.6 ± 8.6	.18

Abbreviations: AJCC = American Joint Committee on Cancer; BMI = body mass index; DFCI = Dana-Farber Cancer Institute; ER = estrogen receptor; HER2− = human epidermal growth factor receptor 2–negative; HER2+ = human epidermal growth factor receptor 2–positive; PR = progesterone receptor; PROMIS = Patient-Reported Outcomes Measurement Information System.

a Mean ± standard deviation for continuous variables and No. (column %) for categorical variables.

b Numbers may not sum to total because of missing data, and percentages may not sum to 100% because of rounding.

c *P* value is for *t*-test (continuous variables), χ^2^ test (categorical variables), or Fisher exact test.

d One Middle Eastern, 1 unknown, and 1 mixed race.

e Three unknown and 6 government-funded insurance.

f Two unknown.

g AJCC 7th edition used for staging first 2 participants only.

h Higher is Better (standardized T-score (mean = 50, SD = 10 in US general population)).

i Higher is Worse (standardized T-score (mean = 50, SD = 10 in US general population)).

**Figure 1.**
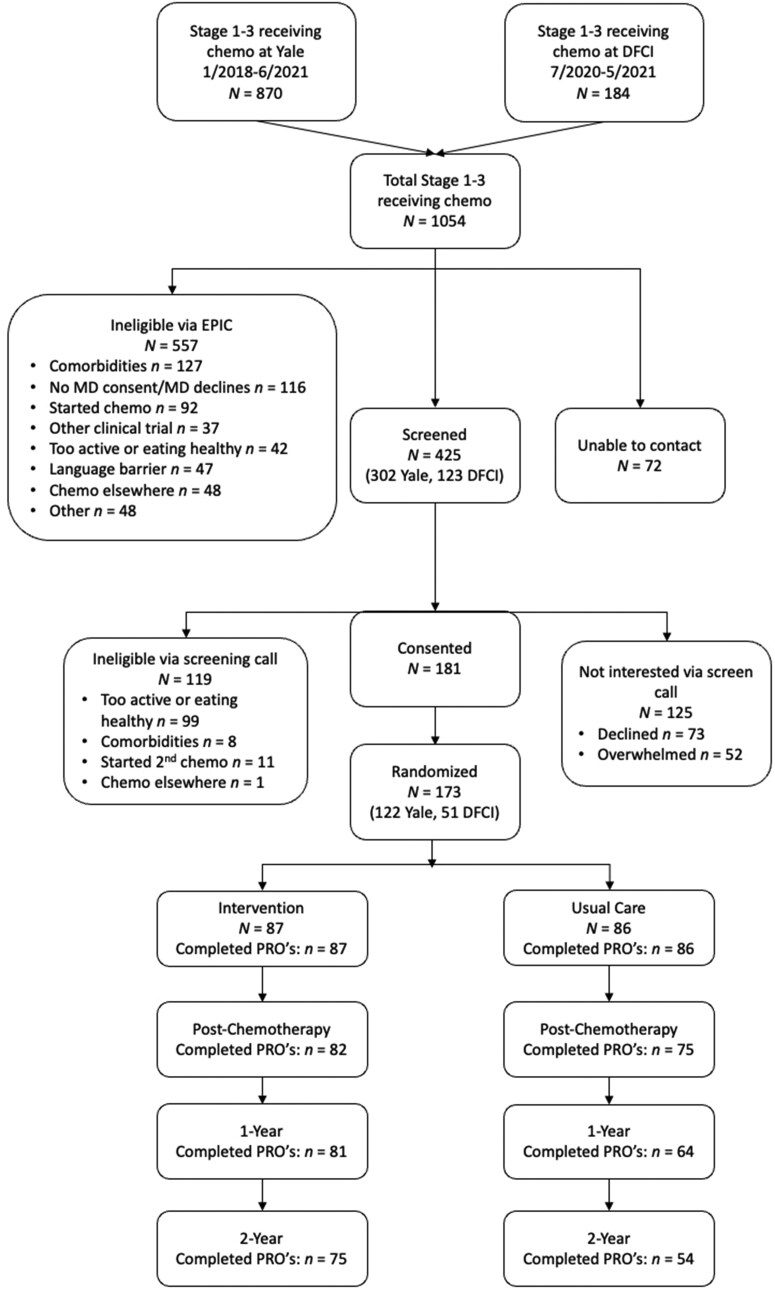
Consort diagram of participants in the Lifestyle, Exercise, and Nutrition Early After Diagnosis study. Abbreviations: DFCI = Dana-Farber Cancer Institute; EPIC = Electronic Health Record System; MD = Doctor of Medicine; PRO’s = patient-reported outcomes.

Of the 173 women randomized, 157 (90.8%) completed PROMIS questionnaires at postchemotherapy, 145 (83.8%) at 1 year, and 129 (74.6%) at 2 years (Figure 1).

### Adherence to intervention

Adherence to the LEANer intervention has been previously reported.^17^^,^^23^ Overall, 90% of women attended ≥80% of the 16 sessions during the first year of diagnosis.^23^ Compared with UC, the intervention arm reported greater increases in exercise minutes at postchemotherapy (mean difference [MD] = 97.6 min/wk, 95% confidence interval [CI] = 63.1 to 132.0; *P* < .001) and 1-year (MD = 136.1 min/wk, 95% CI = 90.2 to 182.0; *P* < .001), and more strength training (postchemotherapy: I = 68% vs UC = 6%; 1-year: I = 56% vs UC = 15%).^23^ Diet quality (HEI score) also improved in the intervention arm compared with UC (postchemotherapy: MD = 3.9 points, 95% CI = 1.0 to6.8; *P* = .01; 1-year: MD = 2.5 points, 95% CI = −0.3 to 5.3; *P* = .08).^23^

### PROMIS-29

**Figure 2.**
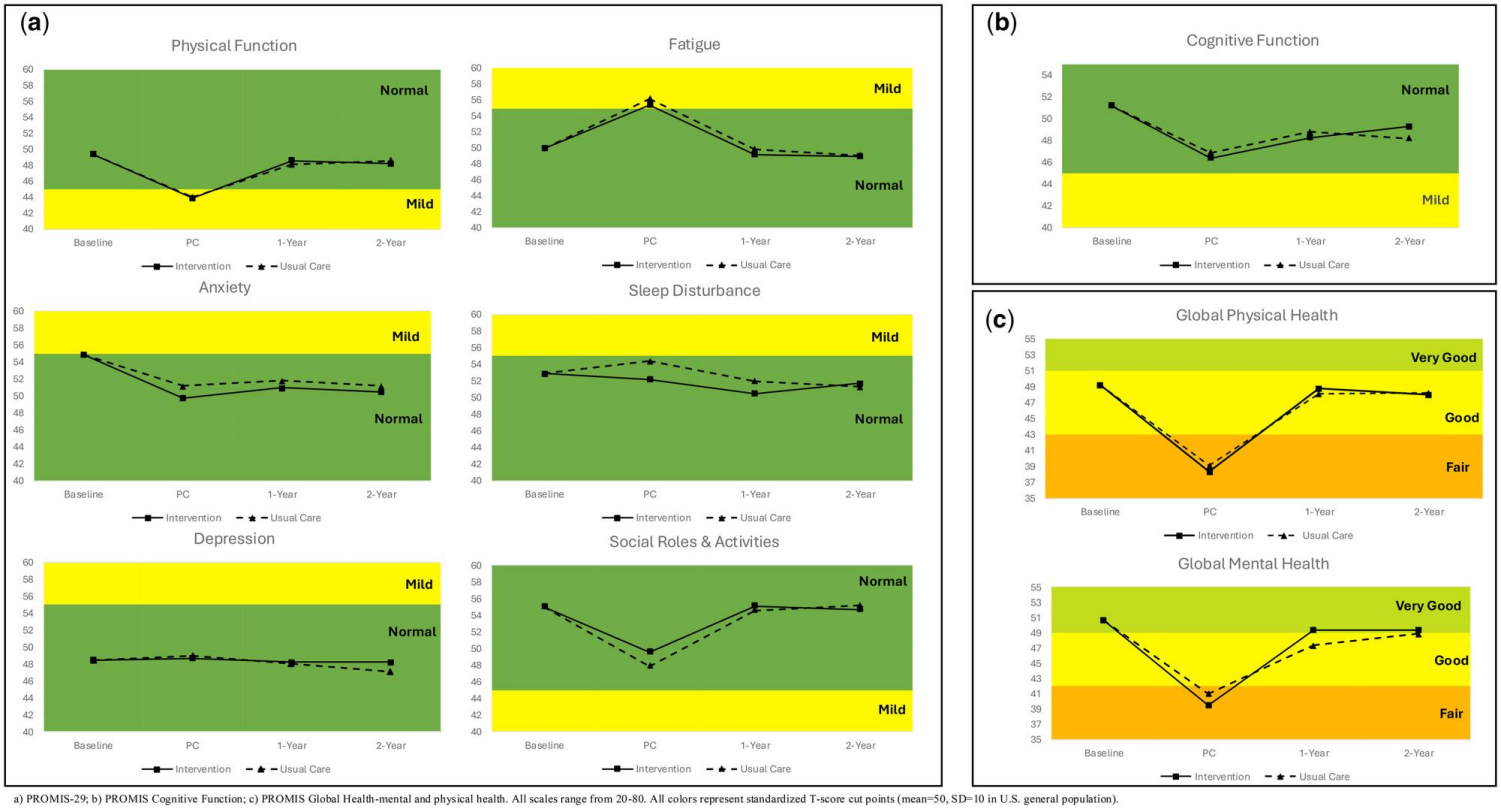
Patient-reported outcomes across study time points by randomization arm. (a) PROMIS-29. (b) PROMIS Cognitive Function. (c) PROMIS Global Health. Abbreviation: PC = postchemotherapy.

At baseline, women reported higher anxiety (54.9; 95% CI: 53.6 to 56.2) and better social roles (55.0; 95% CI = 53.7 to 56.3) than the general population (mean score = 50; SD = 10) (Figure 2A). There were no significant differences by arm for change in PROMIS-29 outcomes at any timepoint (Table 2). Both arms had clinically meaningful worsening in physical function (I = −5.5; 95% CI = −7.4 to −3.6; UC = −5.4; 95% CI = −7.4 to −3.5), fatigue (I = 5.4; 95% CI = 3.3 to 7.6; UC = 6.2; 95% CI = 3.9 to 8.4), and social roles (I = −5.4; 95% CI = −7.4 to −3.4; UC = −7.1; 95% CI = −9.1 to −5.0) but improvements in anxiety (I = −5.1; 95% CI = −6.8 to −3.3; UC = −3.7; 95% CI = −5.6 to −1.9) from baseline to postchemotherapy. Physical function, fatigue, and social roles returned to pretreatment levels for both arms at 1-year and remained stable through 2-years. The improvement in anxiety remained stable through 1-year and 2-years.

**Table 2.** Effect of intervention versus usual care on PROMIS-29, PROMIS Cognitive Function, and PROMIS Global Health at baseline, postchemotherapy, 1-year, and 2-years (N = 173).

	**Intervention** ^a^ **(*n* = 87)**	**Usual care** ^a^ **(*n* = 86)**	**Mean change, least square mean (95% CI)** ^a^	***P*** ^a^
PROMIS-29				
Physical function^c^				
Combined baseline	49.4 (48.1 to 50.6)			
Baseline to PC change	−5.5 (−7.4 to −3.6)	−5.4 (−7.4 to −3.5)	−0.1 (−2.6 to 2.4)	.94
Baseline to 1-year change	−0.8 (−2.6 to 1.1)	−1.3 (−3.2 to 0.7)	0.5 (−2.0 to 2.9)	.70
Baseline to 2-year change	−1.2 (−3.2 to 0.7)	−0.8 (−3.0 to 1.5)	−0.5 (−3.3 to 2.3)	.74
Anxiety^d^				
Combined baseline	54.9 (53.6 to 56.2)			
Baseline to PC change	−5.1 (−6.8 to −3.3)	−3.7 (−5.6 to −1.9)	−1.3 (−3.8 to 1.1)	.28
Baseline to 1-year change	−3.9 (−5.8 to−2.1)	−3.1 (−5.2 to −1.0)	−0.8 (−3.6 to 1.9)	.54
Baseline to 2-year change	−4.4 (−6.4 to −2.5)	−3.7 (−6.0 to −1.5)	−0.7 (−3.6 to 2.2)	.63
Depression^d^				
Combined baseline	48.5 (47.4 to 49.6)			
Baseline to PC change	0.2 (−1.5 to 1.9)	0.5 (−1.3 to 2.2)	−0.2 (−2.5 to 2.0)	.83
Baseline to 1-year change	−0.3 (−1.8 to 1.2)	−0.5 (−2.1 to 1.2)	0.2 (−2.0 to 2.3)	.89
Baseline to 2-year change	−0.3 (−2.1 to 1.5)	−1.4 (−3.4 to 0.6)	1.1 (−1.5 to 3.7)	.42
Fatigue^d^				
Combined baseline	50.0 (48.6 to 51.5)			
Baseline to PC change	5.4 (3.3 to 7.6)	6.2 (3.9 to 8.4)	−0.7 (−3.5 to 2.1)	.61
Baseline to 1-year change	−0.8 (−2.8 to 1.3)	−0.1 (−2.3 to 2.2)	−0.7 (−3.6 to 2.2)	.63
Baseline to 2-year change	−1.0 (−3.3 to 1.2)	−0.9 (−3.4 to 1.7)	−0.2 (−3.4 to 3.0)	.91
Sleep disturbance^d^				
Combined baseline	52.9 (51.6 to 54.3)			
Baseline to PC change	−0.7 (−2.5 to 1.1)	1.5 (−0.4 to 3.3)	−2.2 (−4.5 to 0.2)	.08
Baseline to 1-year change	−2.4 (−4.3 to −0.6)	−0.9 (−3.0 to 1.1)	−1.5 (−4.1 to 1.1)	.25
Baseline to 2-year change	−1.2 (−3.0 to 0.5)	−1.6 (−3.6 to 0.4)	0.4 (−2.1 to 2.9)	.75
Social roles and activities^c^				
Combined baseline	55.0 (53.7 to 56.3)			
Baseline to PC change	−5.4 (−7.4 to −3.4)	−7.1 (−9.1 to −5.0)	1.7 (−0.9 to 4.3)	.20
Baseline to 1-year change	0.1 (−1.7 to 2.0)	−0.4 (−2.4 to 1.7)	0.5 (−2.1 to 3.0)	.72
Baseline to 2-year change	−0.3 (−2.4 to 1.9)	0.2 (−2.2 to 2.7)	−0.5 (−3.6 to 2.6)	.76
PROMIS Cognitive Function				
Cognitive Function^c^				
Combined baseline	51.2 (49.7 to 52.7)			
Baseline to PC change	−4.8 (−6.9 to −2.8)	−4.3 (−6.4 to −2.1)	−0.6 (−3.3 to 2.2)	.69
Baseline to 1-year change	−2.9 (−5.1 to −0.7)	−2.4 (−4.8 to −0.0)	−0.5 (−3.6 to 2.6)	.75
Baseline to 2-year change	−1.9 (−4.2 to 0.3)	−3.0 (−5.6 to −0.4)	1.1 (−2.2 to 4.4)	.52
PROMIS Global Health				
Global Physical Health^c^				
Combined baseline	49.2 (48.1 to 50.2)			
Baseline to PC change^b^	−10.9 (−12.5 to −9.4)	−10.1 (−11.7 to −8.5)	−0.9 (−2.1 to 0.4)	.19
Baseline to 1-year change	−0.4 (−1.9 to 1.1)	−1.1 (−2.7 to 0.6)	0.7 (−1.5 to 2.8)	.54
Baseline to 2-year change	−1.2 (−2.7 to 0.4)	−1.0 (−2.8 to 0.8)	−0.1 (−2.4 to 2.2)	.92
Global Mental Health^c^				
Combined baseline	50.7 (49.5 to 52.0)			
Baseline to PC change^b^	−11.2 (−13.5 to −9.0)	−9.7 (−12.0 to −7.5)	−1.5 (−3.2 to 0.3)	.09
Baseline to 1-year change	−1.3 (−3.1 to 0.5)	−3.3 (−5.3 to −1.3)	2.0 (−0.6 to 4.6)	.13
Baseline to 2-year change	−1.3 (−3.0 to 0.5)	−1.8 (−3.8 to 0.2)	0.5 (−2.0 to 3.1)	.68

Abbreviations: CI = confidence interval; PC = postchemotherapy; PROMIS = Patient-Reported Outcomes Measurement Information System.

a Means, intervention effect, and corresponding *P* values are estimated using a mixed effect model.

b Missing 1 observation, *N* = 81.

c Positive change is improving; negative change is worsening.

d Positive change is worsening, negative change is improving.

### PROMIS Cognitive Function

At baseline, women reported similar cognitive function (51.2; 95% CI = 49.7 to 52.7) compared with the general U.S. population (Figure 2B). There were no significant differences by arm for change in cognitive function at any timepoint (Table 2). Both arms had clinically meaningful worsening in cognitive function (I = −4.8; 95% CI = −6.9 to −2.8; UC = −4.3; 95% CI = −6.4 to −2.1) from baseline to postchemotherapy. Cognitive function improved at 1-year and remained stable through 2-years.

### PROMIS Global Health

At baseline, women reported similar global physical health (49.2; 95% CI = 48.1 to 50.2) and global mental health (50.7; 95% CI = 49.5 to 52.0) compared with the general U.S. population (Figure 2C). There were no significant differences by arm for change in global physical health or global mental health at any timepoint (Table 2). Both arms had clinically meaningful worsening in global physical health (I = −10.9; 95% CI = −12.5 to −9.4; UC = −10.1; 95% CI = −11.7 to −8.5) and global mental health (I = −11.2; 95% CI = −13.5 to −9.0; UC = −9.7; 95% CI = −12.0 to −7.5) from baseline to postchemotherapy. Global physical health and global mental health returned to pretreatment levels for both arms at 1-year and remained stable through 2-years.

### Effect modification

Baseline PROMIS scores, cancer subtype, and chemotherapy drug did not modify group differences in PROMIS scores at all timepoints. Age modified the effect of the intervention on global mental health at postchemotherapy (*P*_interaction_ = .03) and 1-year (*P*_interaction_ = .05). Among women younger than 53, women randomized to intervention reported similar worsening in global mental health as UC from baseline to post-chemotherapy (effect size = 1.8; 95% CI = −1.3 to 4.8, *P* = .25) and baseline to 1-year (effect size = −2.2; 95% CI = −6.5 to 0.1; *P* = .32). Among women older than 53, women randomized to intervention had a greater worsening in global mental health from baseline to postchemotherapy (effect size = −2.9; 95% CI = −5.9 to 0.1; *P* = .06) but recovered to baseline levels at 1-year compared with a decline among UC (effect size = 3.9; 95% CI = −0.3 to 8.1; *P* = .07).

### Baseline factors associated with Post-Chemotherapy changes in PROMIS measures

Compared with women with a college degree or above, women with less than a college degree had greater worsening in fatigue (least squared mean ± standard error = 3.8 ± 1.9; *P* = .04) (Table 3). Compared with adjuvant chemotherapy, women treated with neoadjuvant chemotherapy reported worsening social roles (−5.4 ± 1.7; *P* = .002), physical function (−6.1 ± 1.5; *P* < .001), physical health (−4.0 ± 1.6; *P* = .01) and mental health (−5.4 ± 2.3; *P* = .02). Compared with stage III, women with stage I and stage II reported worsening physical function (−7.4 ± 2.3, −4.5 ± 2.4 respectively; *P* = .004). Older age was associated with significant worsening in social roles (*P* = .03), physical function (*P* = .001), physical health (*P* = .003), and mental health (*P* = .04) but improvement in cognitive function (*P* = .02). Higher BMI was associated with worsening in physical function (*P* = .002), cognitive function (*P* = .03) but improvement in physical health (*P* = .001).

**Table 3.** Factors associated with changes in PROMIS measures at postchemotherapy.

	**PROMIS-29** ^a^								PROMIS Cognitive		PROMIS Global Health			
	**Anxiety** ^b^		**Fatigue** ^b^		**Social roles** ^b^		**Physical function** ^b^		**Cognitive** ^b^		**Physical Health** ^b^		**Mental Health** ^b^	
	Estimate (SE)	*P*	Estimate (SE)	*P*	Estimate (SE)	*P*	Estimate (SE)	*P*	Estimate (SE)	*P*	Estimate (SE)	*P*	Estimate (SE)	*P*
Age (y)	−0.10 (0.1)	.31	0.22 (0.1)	.06	−0.24 (0.1)	.03	−0.32 (0.1)	.001	0.26 (0.1)	.02	−0.31 (0.1)	.003	−0.32 (0.1)	.04
BMI (kg/m^2^)	0.02 (0.1)	.89	0.14 (0.1)	.29	−0.16 (0.1)	.19	−0.33 (0.1)	.002	−0.26 (0.1)	.03	0.44 (0.1)	.001	0.15 (0.2)	.37
Chemotherapy														
Neoadjuvant	−0.97 (1.5)	.52	1.16 (1.8)	.53	−5.42 (1.7)	.002	−6.06 (1.5)	<.001	1.88 (1.7)	.28	−3.96 (1.6)	.01	−5.44 (2.3)	.02
Adjuvant	Referent		Referent		Referent		Referent		Referent		Referent		Referent	
Cancer stage														
Stage I	1.80 (2.3)	.73	−1.08 (2.9)	.70	−4.80 (2.7)	.18	−7.42 (2.3)	.004	−3.99 (2.7)	.29	1.29 (2.5)	.87	3.96 (3.6)	.19
Stage II	1.12 (2.4)		−2.30 (3.0)		−4.60 (2.8)		−4.53 (2.4)		−4.17 (2.8)		1.11 (2.5)		−0.34 (3.8)	
Stage III	Referent		Referent		Referent		Referent		Referent		Referent		Referent	
Self-report race and ethnicity														
Non-Hispanic, White	0.69 (3.0)	.65	−0.80 (3.7)	.99	3.03 (3.4)	.41	0.64 (3.0)	.72	−0.71 (3.4)	.79	−0.19 (3.1)	.82	−7.09 (4.6)	.12
Non-Hispanic, Black	1.74 (3.4)		−0.01 (4.2)		4.25 (3.9)		3.04 (3.4)		−0.51 (3.9)		−2.12 (3.6)		−12.70 (5.4)	
Hispanic	−2.58 (4.0)		−0.23 (4.9)		7.26 (4.5)		0.86 (3.9)		2.71 (4.6)		−1.97 (4.2)		−6.07 (6.2)	
Other	Referent		Referent		Referent		Referent		Referent		Referent		Referent	
Living with someone														
Married/living with someone	Referent	.65	Referent	.92	Referent	.96	Referent	.80	Referent	.71	Referent	.77	Referent	.26
Living alone	0.76 (1.7)		−0.19 (2.0)		0.08 (1.9)		−0.42 (1.6)		−0.71 (1.9)		−0.51 (1.7)		2.89 (2.6)	
Education level														
Less than college	2.60 (1.5)	.09	3.83 (1.9)	.04	0.71 (1.7)	.68	0.19 (1.5)	.90	0.16 (1.7)	.93	1.10 (1.6)	.50	1.02 (2.4)	.67
College or above	Referent		Referent		Referent		Referent		Referent		Referent		Referent	
Menopausal status														
Premenopausal	−0.46 (2.1)	.81	−0.48 (2.6)	.47	−1.96 (2.4)	.43	−3.37 (2.1)	.19	1.39 (2.4)	.50	−1.63 (2.2)	.74	−2.46 (3.3)	.75
Postmenopausal	Referent		Referent		Referent		Referent		Referent		Referent		Referent	

Abbreviations: BMI = body mass index; PROMIS = Patient-Reported Outcomes Measurement Information System; SE = standard error.

a Reporting only PROMIS-29 domains with clinically meaningful changes (3-T score) from baseline to postchemotherapy (ie, did not include PROMIS-29 Sleep Disturbance and PROMIS-29 Depression).

b Models adjusted for variables significantly different at *P* < .1 level in univariate models.

### Correlations between changes in exercise and diet quality and changes in PROMIS measures

Exploratory analyses showed weak but significant correlations between increases in exercise from baseline to postchemotherapy and improvements in social roles (*r *= 0.21, *P* = .01). Increases in exercise from baseline to 1-year were weakly correlated with reductions in anxiety and depression (*r* = −0.20, *P* = .02 and *r* = −0.25, *P* = .003, respectively) and improved mental health (*r *= 0.22, *P* = .01). Improvements in diet quality from baseline to 1-year were weakly correlated with reductions in sleep disturbance (*r* = −0.22, *P* = .01).

## Discussion

Substantial research has informed clinical guidelines supporting exercise during cancer treatment to preserve physical function and reduce fatigue, anxiety and depression.^13^ However, while nutrition is widely recognized as important during chemotherapy, there is limited research on nutrition interventions during treatment, either alone or combined with exercise, making it difficult to provide clear clinical recommendations.^13^^,^^38^ In this secondary analysis of the LEANer yearlong nutrition and exercise intervention among women initiating curative-intent chemotherapy for breast cancer, both intervention and UC groups reported worsening in the QoL measures—physical function, fatigue, social roles, cognitive function, and global health—at completion of chemotherapy, with no between-arm differences.

Our findings are inconsistent with studies reporting small but significant benefits of exercise during treatment on fatigue, physical function, and QoL.^10^^,^^39^^,^^40^ Several factors may explain these findings. First, the intensity of the LEANer intervention may not have been enough to impact QoL. Despite the intervention leading to a significant self-reported improvement in exercise and diet quality compared with UC, we did not objectively assess physical activity, and validated objective measures of diet quality do not exist.^23^ Second, the intervention was unsupervised and home-based, with weekly calls from RDs. Previous research suggests supervised exercise programs during and after treatment may impact QoL and physical function more than unsupervised sessions.^10^ Third, a hypothesized mechanism that could drive improved QoL was through reduced treatment-related side effects, particularly nutrition impact symptoms. We previously reported that 54.9% of women randomized to intervention reported any severe nutrition impact symptoms versus 62.7% of women randomized to usual care (*P* = .32).^17^ Last, to our knowledge, no other trials during chemotherapy have assessed QoL using PROMIS measures. Thus, it is possible that PROMIS instruments are not sensitive enough to capture small but clinically relevant changes in QoL during this phase of cancer care.

All participants experienced declines in mental health during treatment, but at 1-year, only older women randomized to intervention returned to pretreatment mental health levels, highlighting the need to develop and test interventions tailored to support the mental health of younger women. In other exploratory analyses, we found women with less education reported greater worsening in fatigue during chemotherapy compared with women with higher education, suggesting socioeconomic factors may play an important role in treatment-related QoL. Neoadjuvant chemotherapy was also associated with worse physical and mental health at postchemotherapy compared with adjuvant chemotherapy, potentially due to neoadjuvant patients still anticipating surgery at that time.

To our knowledge, only 2 observational studies have assessed PROMIS domains at diagnosis and during treatment for breast cancer.^41^^,^^42^ One retrospective study of 215 breast cancer patients reported similar baseline global mental and physical health as our trial, with declines during chemotherapy and recovery afterward.^42^ We observed a greater decline during treatment, possibly due the timing of our assessment being immediately after chemotherapy when treatment-related symptoms are at their peak. Another study of 2746 women with breast cancer before treatment, found worse physical function, greater fatigue, and similar anxiety compared with our cohort.^41^ Higher symptom burden in this study may have been due to administering certain questionnaires only if a patient reported impairment on a screening question.^41^ Across studies, most PROMIS measures appear to be similar to the US population at diagnosis, though our study and Matthys et al.^41^ reported elevated anxiety at diagnosis. This is likely due to anticipatory effects of chemotherapy, though may improve once treatment begins, as seen in our cohort. While the clinical implications of transient declines in PROMIS domains remain unclear, below average PROMIS physical and mental health scores after breast cancer treatment have been associated with higher risk of all-cause mortality.^43^

This study is among the first to evaluate a combined nutrition and exercise intervention during chemotherapy for breast cancer on PROMIS QoL outcomes. Strengths include an intervention aligned with lifestyle guidelines and tailored for women undergoing chemotherapy. Adherence to intervention sessions was high and participants reported significant increases in physical activity and diet quality. We collected a comprehensive set of PROMIS measures, providing data on more domains than prior observational studies and examined changes longitudinally. However, PROMIS measures were collected only at postchemotherapy, 1-year and 2-years, which may not capture symptom fluctuations across chemotherapy cycles. More frequent collection of PROMIS measures may better evaluate short-term changes in these outcomes. We did not collect real-time use of anti-nausea or other supportive medications, which may have differed between groups, potentially contributing to the null findings. The UC group also had access to the Survivorship Clinic and could request a nutrition consultation as standard of care, potentially introducing contamination. In addition, this secondary analysis may have been underpowered for these outcomes. Finally, the study populations consisted of primarily highly educated, non-Hispanic White, English-speaking population of women with early-stage breast cancer, potentially limiting generalizability and clinical applicability of our findings.

Maintaining QoL during and after chemotherapy is a critical component of oncology care. Although this intervention did not attenuate declines in QoL compared with UC, these findings provide novel evidence and raise important considerations for future research. Additional research is necessary to understand the best timing to assess patient-reported outcomes, the optimal intervention dose and delivery, and to identify for whom nutrition and exercise interventions during treatment can attenuate declines in treatment-related QoL.

## Data Availability

All individual participant data collected during the trial, after deidentification, can be made available by the corresponding author upon reasonable request. In addition, the study Protocol, statistical analysis plan, informed consent form, and analytic code can also be shared. These data can be shared immediately after publication with no end date.

## References

[1] Guimond A-J , Ivers H, Savard J. Clusters of psychological symptoms in breast cancer: is there a common psychological mechanism? Cancer Nurs. 2020;43:343-353. 10.1097/ncc.0000000000000705 30950932

[2] Montgomery GH , David D, Goldfarb AB, et al Sources of anticipatory distress among breast surgery patients. J Behav Med. 2003;26:153-164. 10.1023/A : 1023034706298 12776384 10.1023/a:1023034706298

[3] Janz NK , Mujahid M, Chung LK, et al Symptom experience and quality of life of women following breast cancer treatment. J Womens Health (Larchmt). 2007;16:1348-1361. 10.1089/jwh.2006.0255 18001192

[4] Ni F , Cai T, Zhou T, Yuan C. Identification of subgroups of self-reported outcomes among breast cancer patients undergoing surgery and chemotherapy: a cross-sectional study. Int J Nurs Sci. 2025;12:51-58. 10.1016/j.ijnss.2024.12.007 39990984 PMC11846581

[5] Lin HP , Kuo YH, Tai WY, Liu HE. Exercise effects on fatigue in breast cancer survivors after treatments: a systematic review and meta-analysis. Int J Nurs Pract. 2022;28:e12989. 10.1111/ijn.12989 34258817

[6] Lake B , Damery S, Jolly K. Effectiveness of weight loss interventions in breast cancer survivors: a systematic review of reviews. BMJ Open. 2022;12:e062288. 10.1136/bmjopen-2022-062288 PMC9557263 36207046

[7] Aune D , Markozannes G, Abar L, et al Physical activity and health-related quality of life in women with breast cancer: a meta-analysis. JNCI Cancer Spectr. 2022;6:pkac072. 10.1093/jncics/pkac072 36474321 PMC9727071

[8] Lee J , Hwang Y. The effects of exercise interventions on fatigue, body composition, physical fitness, and biomarkers in breast cancer patients during and after treatment: a systematic review and meta-analysis of randomized controlled trials. J Cancer Surviv. 2025;1-29. 10.1007/s11764-025-01772-x 40056311

[9] Cadmus LA , Salovey P, Yu H, Chung G, Kasl S, Irwin ML. Exercise and quality of life during and after treatment for breast cancer: results of two randomized controlled trials. Psychooncology. 2009;18:343-352. 10.1002/pon.1525 19242918 PMC4221990

[10] Sweegers MG , Altenburg TM, Chinapaw MJ, et al Which exercise prescriptions improve quality of life and physical function in patients with cancer during and following treatment? A systematic review and meta-analysis of randomised controlled trials. Br J Sports Med. 2018;52:505-513. 10.1136/bjsports-2017-097891 28954800

[11] van Waart H , Stuiver MM, van Harten WH, et al Effect of low-intensity physical activity and moderate- to high-intensity physical exercise during adjuvant chemotherapy on physical fitness, fatigue, and chemotherapy completion rates: results of the PACES randomized clinical trial. J Clin Oncol. 2015;33:1918-1927. 10.1200/jco.2014.59.1081 25918291

[12] Courneya KS , Segal RJ, Mackey JR, et al Effects of aerobic and resistance exercise in breast cancer patients receiving adjuvant chemotherapy: a multicenter randomized controlled trial. J Clin Oncol. 2007;25:4396-4404. 10.1200/jco.2006.08.2024 17785708

[13] Ligibel JA , Bohlke K, May AM, et al Exercise, diet, and weight management during cancer treatment: ASCO guideline. J Clin Oncol. 2022;40:2491-2507. 10.1200/jco.22.00687 35576506

[14] Salas S , Cottet V, Dossus L, et al Nutritional factors during and after cancer: impacts on survival and quality of life. Nutrients. 2022;14:2958. 10.3390/nu14142958 35889914 PMC9323157

[15] Barchitta M , Maugeri A, Magnano San Lio R, et al The effects of diet and dietary interventions on the quality of life among breast cancer survivors: a cross-sectional analysis and a systematic review of experimental studies. Cancers (Basel). 2020;12:322. 10.3390/cancers12020322 32019093 PMC7072135

[16] Parsons HM , Forte ML, Abdi HI, et al Nutrition as prevention for improved cancer health outcomes: a systematic literature review. JNCI Cancer Spectr. 2023;7:pkad035. 10.1093/jncics/pkad035 37212631 PMC10290234

[17] Sanft T , Harrigan M, McGowan C, et al Randomized trial of exercise and nutrition on chemotherapy completion and pathologic complete response in women with breast cancer: the lifestyle, exercise, and nutrition early after diagnosis study. J Clin Oncol. 2023;41:5285-5295. 10.1200/jco.23.00871 37656930 PMC10691793

[18] Tong H , Isenring E, Yates P. The prevalence of nutrition impact symptoms and their relationship to quality of life and clinical outcomes in medical oncology patients. Support Care Cancer. 2009;17:83-90. 10.1007/s00520-008-0472-7 18551322

[19] Sanft T , Harrigan M, Cartmel B, et al Effect of healthy diet and exercise on chemotherapy completion rate in women with breast cancer: the Lifestyle, Exercise and Nutrition Early after Diagnosis (LEANer) study: study protocol for a randomized clinical trial. Contemp Clin Trials. 2021;109:106508. 10.1016/j.cct.2021.106508 34274495 PMC10424280

[20] Harrigan M , Cartmel B, Loftfield E, et al Randomized trial comparing telephone versus in-person weight loss counseling on body composition and circulating biomarkers in women treated for breast cancer: the Lifestyle, Exercise, and Nutrition (LEAN) study. J Clin Oncol. 2016;34:669-676. 10.1200/jco.2015.61.6375 26598750 PMC4872022

[21] The Diabetes Prevention Program (DPP): description of lifestyle intervention. Diabetes Care. 2002;25:2165-2171. 10.2337/diacare.25.12.2165 12453955 PMC1282458

[22] Bandura A. Social cognitive theory in cultural context. Appl Psychol. 2002;51:269-290.

[23] Puklin LS , Ferrucci LM, Harrigan M, et al Improving lifestyle behaviors during chemotherapy for breast cancer: the Lifestyle, Exercise, and Nutrition Early After Diagnosis (LEANer) trial. Cancer. 2024;130:2440-2452. 10.1002/cncr.35280 38470431 PMC11214600

[24] Piercy KL , Troiano RP, Ballard RM, et al The Physical Activity Guidelines for Americans. JAMA. 2018;320:2020-2028. 10.1001/jama.2018.14854 30418471 PMC9582631

[25] Rock CL , Thomson CA, Sullivan KR, et al American Cancer Society nutrition and physical activity guideline for cancer survivors. CA Cancer J Clin. 2022;72:230-262. 10.3322/caac.21719 35294043

[26] Campbell KL , Winters-Stone KM, Wiskemann J, et al Exercise guidelines for cancer survivors: consensus statement from international multidisciplinary roundtable. Med Sci Sports Exerc 2019;51:2375-2390. 31626055 10.1249/MSS.0000000000002116 PMC8576825

[27] Services USDoAaUSDoHaH. Dietary Guidelines for Americans, 2020-2025. DietaryGuidelines.gov

[28] Kriska AM , Knowler WC, LaPorte RE, et al Development of questionnaire to examine relationship of physical activity and diabetes in Pima Indians. Diabetes Care. 1990;13:401-411. 10.2337/diacare.13.4.401 2318100

[29] Nutrition Assessment. Fred Hutch Cancer Center. Accessed May, 16, 2025. https://www.fredhutch.org/en/research/divisions/public-health-sciences-division/research/nutrition-assessment.html

[30] Schakel SF. Maintaining a nutrient database in a changing marketplace: keeping pace with changing food products—a research perspective. J Food Comp Anal. 2001;14:315-322. 10.1006/jfca.2001.0992

[31] Krebs-Smith SM , Pannucci TE, Subar AF, et al Update of the Healthy Eating Index: HEI-2015. J Acad Nutr Diet. 2018;118:1591-1602. 10.1016/j.jand.2018.05.021 30146071 PMC6719291

[32] Cella D , Choi SW, Condon DM, et al PROMIS^®^ adult health profiles: efficient short-form measures of seven health domains. Value Health. 2019;22:537-544. 10.1016/j.jval.2019.02.004 31104731 PMC7201383

[33] Iverson GL , Marsh JM, Connors EJ, Terry DP. Normative reference values, reliability, and item-level symptom endorsement for the PROMIS^®^ v2.0 cognitive function-short forms 4a, 6a and 8a. Arch Clin Neuropsychol. 2021;36:1341-1349. 10.1093/arclin/acaa128 33454756

[34] Hays RD , Bjorner JB, Revicki DA, Spritzer KL, Cella D. Development of physical and mental health summary scores from the Patient-Reported Outcomes Measurement Information System (PROMIS) global items. Qual Life Res. 2009;18:873-880. 10.1007/s11136-009-9496-9 19543809 PMC2724630

[35] HealthMeasures. HealthMeasures Scoring Service powered by Assessment Center. Accessed October 23, 2024. https://www.assessmentcenter.net/ac_scoringservice

[36] Jensen RE , Moinpour CM, Potosky AL, et al Responsiveness of 8 Patient-Reported Outcomes Measurement Information System (PROMIS) measures in a large, community-based cancer study cohort. Cancer. 2017;123:327-335. 10.1002/cncr.30354 27696377 PMC5222745

[37] Yost KJ , Eton DT, Garcia SF, Cella D. Minimally important differences were estimated for six Patient-Reported Outcomes Measurement Information System-Cancer scales in advanced-stage cancer patients. J Clin Epidemiol. 2011;64:507-516. 10.1016/j.jclinepi.2010.11.018 21447427 PMC3076200

[38] Schmitz KH , Brown JC, Irwin ML, et al; ENICTO Consortium. Exercise and Nutrition to Improve Cancer Treatment-Related Outcomes (ENICTO). J Natl Cancer Inst 2025;117:9-19. 10.1093/jnci/djae177 39118255 PMC11717426

[39] Ehlers DK , DuBois K, Salerno EA. The effects of exercise on cancer-related fatigue in breast cancer patients during primary treatment: a meta-analysis and systematic review. Expert Rev Anticancer Ther. 2020;20:865-877. 10.1080/14737140.2020.1813028 32842816

[40] Juvet LK , Thune I, Elvsaas I, et al The effect of exercise on fatigue and physical functioning in breast cancer patients during and after treatment and at 6 months follow-up: a meta-analysis. Breast. 2017;33:166-177. 10.1016/j.breast.2017.04.003 28415013

[41] Matthys MB , Dempsey AM, Melisko ME, et al Incorporation of Patient-Reported Outcomes Measurement Information System to assess quality of life among patients with breast cancer initiating care at an academic center. Cancer. 2021;127:2342-2349. 10.1002/cncr.33496 33957704

[42] Azad AD , Yilmaz M, Bozkurt S, Brooks JD, Blayney DW, Hernandez-Boussard T. Diverse patient trajectories during cytotoxic chemotherapy: capturing longitudinal patient-reported outcomes. Cancer Med. 2021;10:5783-5793. 10.1002/cam4.4124 34254459 PMC8419778

[43] Park J , Rodriguez JL, O’Brien KM, et al Health-related quality of life outcomes among breast cancer survivors. Cancer. 2021;127:1114-1125. 10.1002/cncr.33348 33237602 PMC8035208

